# The Impact of Experience on Affective Responses during Action Observation

**DOI:** 10.1371/journal.pone.0154681

**Published:** 2016-05-05

**Authors:** Louise P. Kirsch, Arielle Snagg, Erin Heerey, Emily S. Cross

**Affiliations:** 1 Wales Institute for Cognitive Neuroscience, School of Psychology, Bangor University, Bangor, Wales; 2 Research Department of Clinical, Educational and Health Psychology, University College London, London, United Kingdom; 3 Department of Neuroscience, Pomona College, Claremont, California, United States of America; 4 Department of Psychology, Western University, London, Ontario, Canada; 5 Department of Social and Cultural Psychology, Behavioural Science Institute, Donders Institute for Brain, Cognition and Behaviour, Radboud University Nijmegen, The Netherlands; VU University Amsterdam, NETHERLANDS

## Abstract

Perceiving others in action elicits affective and aesthetic responses in observers. The present study investigates the extent to which these responses relate to an observer’s general experience with observed movements. Facial electromyographic (EMG) responses were recorded in experienced dancers and non-dancers as they watched short videos of movements performed by professional ballet dancers. Responses were recorded from the *corrugator supercilii (CS)* and *zygomaticus major (ZM)* muscles, both of which show engagement during the observation of affect-evoking stimuli. In the first part of the experiment, participants passively watched the videos while EMG data were recorded. In the second part, they explicitly rated how much they liked each movement. Results revealed a relationship between explicit affective judgments of the movements and facial muscle activation only among those participants who were experienced with the movements. Specifically, CS activity was higher for disliked movements and ZM activity was higher for liked movements among dancers but not among non-dancers. The relationship between explicit liking ratings and EMG data in experienced observers suggests that facial muscles subtly echo affective judgments even when viewing actions that are not intentionally emotional in nature, thus underscoring the potential of EMG as a method to examine subtle shifts in implicit affective responses during action observation.

## Introduction

Perceiving others in action, whether in daily contexts like seeing a commuter run to catch a train, or in highly refined artistic settings, such as watching a skilled dancer perform on stage, evokes both explicit and implicit affective responses in observers. Quantifying observers’ affective experiences presents an experimental challenge, as such evaluations are subjective and shaped by many factors, including experience, expectations, and context [1]. Most studies to date that have investigated this topic have relied on participants’ explicit, self-reported affective experience with a stimulus [2]. However, self-reports are prone to memory and response biases that can compromise accuracy [3]. Moreover, the act of reporting an affective experience engages participants in a secondary self-reflective task that has the potential to contaminate both overt behavioural results and covert measures of affective experience, such as neuroimaging evidence [2].

One method that offers a promising way to circumvent the issues associated with subjective self-report is facial electromyography (EMG), which provides a sensitive means to measure observers’ affective experience [4]. This technique measures electrical activity related to muscle tension and can assess subtle expressions of emotion, such as those associated with different affective states. Importantly, EMG measures offer the ability to capture minute shifts in emotion or affective state that may not reach an observer’s threshold for self-report, meaning that EMG may offer a way to measure nascent valenced evaluations of complex stimuli even when they do not have obvious (or yet-discernable) affective elements. An established literature documents the use of facial EMG to measure emotion, based on its ability to detect subtle changes in affective responses in facial muscles across time [5–11]. As previously suggested by Darwin [12], the face is central to the experience of emotion. Thus, facial expressions, such as smiles or frowns, are key indicators of affective experience in humans.

A number of facial EMG studies have explored observers’ physiological responses when viewing faces with emotional expressions [13–16]. This work shows that when people view emotional facial expressions, they spontaneously produce corresponding or complementary facial displays that can be discriminated based on distinct EMG activity patterns. For example, the *corrugator supercilii* (CS) muscle is engaged during frowning and is associated with perceiving negatively-valenced stimuli, whereas the *zygomaticus major* (ZM) is active during smiling and is engaged when perceiving positive stimuli [17]. Recently, Grèzes and colleagues [18] generalized previous findings on the observation of emotional facial expressions to dynamic displays of emotional body expressions. They demonstrated that watching a body express anger induces greater *corrugator supercilii* activity in the observer compared to watching a body moving neutrally. These findings suggest that observing others’ body movements can also evoke emotional facial responses in observers when stimuli are clearly emotionally valenced. In the present study, we aimed to extend this past work by examining how the human face responds to bodies performing a broader range of actions that are not intentionally or overtly emotionally-valenced, and may thus elicit a range of affective responses. Specifically, we ask whether complex dance actions that are not specifically emotional in nature might nonetheless evoke pleasure and displeasure among skilled and novice observers, and whether these responses correspond with differential engagement of facial muscles, as measured by EMG.

Our interest in probing the relationship between experience and affective facial responses stems in part from prior research into the impact of an observer’s past experience on aesthetic appraisal. Studies using behavioural, physiological (such as galvanic skin response–GSR) or neuroimaging approaches have demonstrated a positive relationship between physical expertise and an observer’s aesthetic evaluation (which is a particular kind of affective judgment) [19–24]. A growing literature documents how aesthetic appraisal of a stimulus draws on affective or emotional processing [25–26], but an important limitation in these prior studies is that participants’ evaluations of stimuli are almost always assessed via subjective self-report ratings (i.e., “on a 1–5 scale, how much do you like this painting?” or “please assign this sculpture a rating of like, dislike or neutral”; though see [24] for a different approach using GSR). It therefore remains to be determined whether implicit affective judgments (as expressed by the face and measured via EMG activity) show similar patterns as self-report responses among individuals who have prior experience with the observed actions, and whether this occurs in individuals without such experience.

In this study, we focus on classical ballet dance movements, which form the backbone of a classically trained dancer’s repertoire and are therefore highly familiar to those with this kind of dance experience. Classical ballet movements are ideal in this context because they do not necessarily depict overt emotional displays [1, 27–28], which evidence shows biases facial reactions [14, 16, 29]. We recorded facial EMG from the CS and ZM muscles in both experienced dancers and non-dancers, and predicted greater CS activity when participants watched movements they disliked and greater ZM activity when watching movements they liked. As our experienced dancer population included dancers specifically experienced in classical ballet, and experienced dancers without a classical ballet background, we are also able to investigate the extent to which prior training of particular movements shapes affective responses, compared to effects that generalize to dance experience more broadly defined [20–21, 30]. As these recent behavioural studies demonstrate, dance experience can enhance affective responses and liking of familiar movements. It is important to note that in the present experiment, we cannot precisely define the type of cognitive appraisal that takes place during action observation. However, our approach does enable us to address how general differences in experience with a specific movement vocabulary shapes engagement of facial muscles that have been linked to affective processing.

## Materials and Methods

### Participants

Fifty-one volunteers from Bangor University and the local community participated in this experiment in exchange for money (£6) or course credit. All participants reported being in good physical and neurological health. Participants were classified as either non-dancers (26 participants, 16 females, *M*_*age*_ = 21.07 years; *SD* = 3.67) or dancers (with at least one year of regular dance practice—at least one class per week; 25 participants, 25 females, *M*_*age*_ = 24.12 years, *SD* = 6.31)–for more details see Table 1. Based on responses to a screening questionnaire, non-dancers reported being “poor” dancers and reported little experience with observing dance, attending an average of <1 professional dance performances per year, whereas participants with dance experience reported being intermediate to good at dancing and reported attending between 1 and 5 dance performances per year. Bangor University’s Ethics Committee approved all experimental procedures and participants provided written informed consent before experimental procedures began.

**Table 1:** Summary of participants demographic and dance experience.

	Non-Dancers	Dancers		
		All Dancers	Non-Ballet Dancers	Ballet Dancers
**Number of participant *(N***_***females***_***)***	26 *(15)*	25 *(25)*	11	14
**Mean age *(SD)***	21.07 *(3*.*67)*	24.12 *(6*.*31)*	24.36 *(7*.*24)*	24 *(5*.*49)*
**Dance experience *(SD)***	< 6 months	> 1 year	6.27 years *(6*.*91)*	8.96 years *(6*.*86)*
**Type of dance**		All types of dance	Dance experience ranged from bharata natyam to Irish step dance to tap and ballroom dance, but little or no ballet training (maximum ballet experience < 6 months).	All ballet dancers reported a minimum of 2 years of ballet training, and reported ballet as their primary dance style if they reported experience with other forms of dance (such as jazz or tap)

Details of participants’ demographic and dance experience. The experienced dancer group has been split into two sub-groups (non-ballet and ballet dancers) for finer analysis of the impact of dance experience on implicit affective responses (see final analysis in the results section). Note no difference in terms of years of dance experience between the two groups of dancers (non-ballet and ballet dancers; p > 0.3).

### Stimuli and Procedure

Stimuli featured a male or female dancer performing a single dance movement. The dancers, both members of the Leipzig Ballet, performed a range of movements varying in complexity, speed, difficulty, and amplitude from both classical ballet and modern ballet dance vocabularies. Forty different stimuli were constructed, each 3s in length. These sequences were used and validated in a previous experiment [19]. From the sixty-four stimuli used in Cross et al.’s study [19], we selected the twenty ‘least’ liked and twenty ‘most’ liked movements, and ensured that each stimulus video featured one whole movement (e.g., a pirouette starting and ending with the dancer standing in first position).

**Fig 1:**
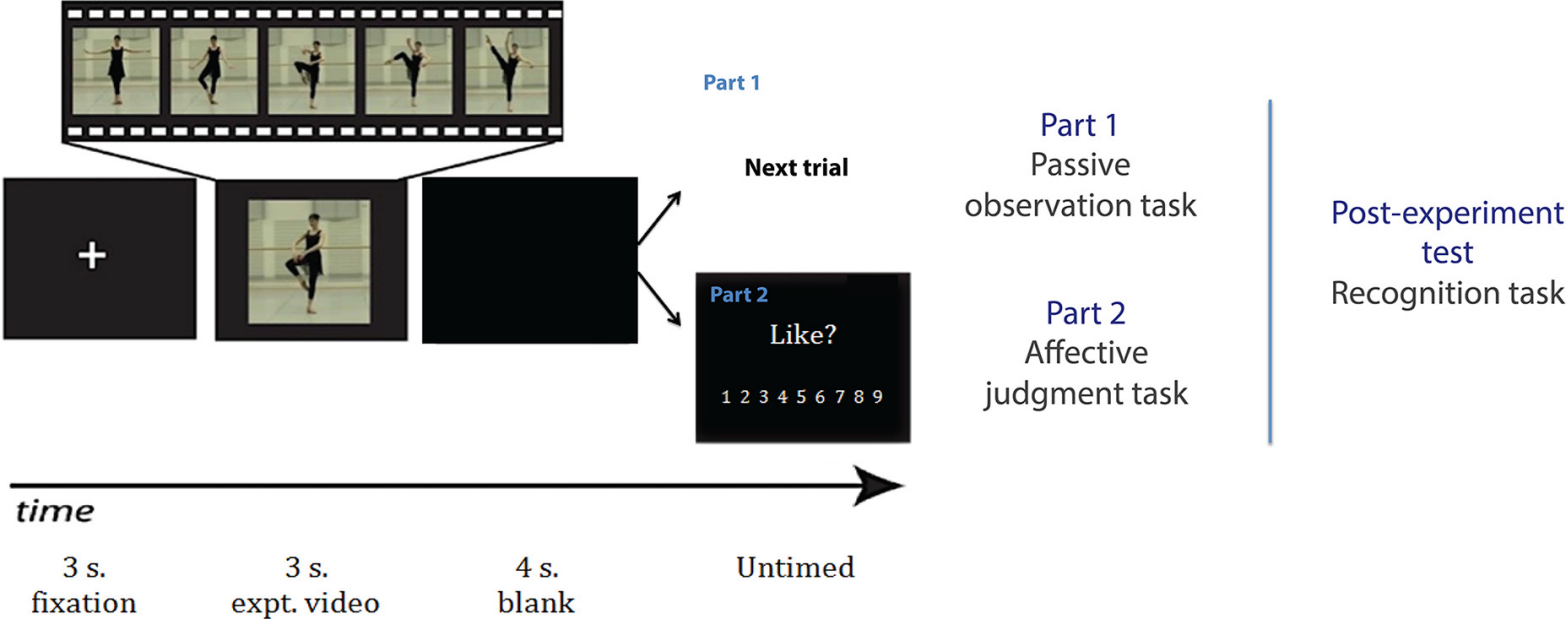
Experimental design. Trials began with a fixation cross that appeared for 3s, followed by a 3s dance sequence and a blank screen for 4s. In Part 1, participants watched the sequences passively, without making any ratings. In Part 2, participants watched each sequence and then rated how much they liked the movement they had viewed. The next trial began after the computer registered a rating. After part 2, participants performed a recognition task, where they watched a number of previously seen and novel dance videos, which they had to decide whether they had seen during the previous task. The dancer whose image appears in the figure consented to the use of her image in research materials and resulting publications.

The present experiment consisted of two main parts. Part 1 comprised a passive observational task and Part 2 comprised an active aesthetic evaluation task (Fig 1). Importantly, we followed the same approach as Cannon, Hayes and Tipper [31], measuring EMG responses before explicit ratings to avoid any contamination of the EMG signal that might be a consequence of making explicit affective judgments. During Part 1, participants simply watched and attended to the dance clips. Trials began with the presentation of a central fixation cross (3s long; see Fig 1), followed by the video clip (3s). All trials during this phase concluded with a blank screen (4s). Participants watched each stimulus passively and did not perform any secondary task. They were instructed to watch each movement closely in order to perform a recognition task at the end of the experiment. Participants completed three blocks of 40 passive viewing trials, randomly ordered. Thus, participants passively viewed each stimulus three times during Part 1. We recorded EMG only during Part 1 of the task.

Part 2 was similar to Part 1, except that participants provided a rating after watching each video. After a short, 8-trial practice block (with novel videos), participants rated each of the 40 stimuli, in random order. Trials began with central fixation, followed by the video and blank screen. Thereafter, the following question appeared on the screen: “How much did you like the previous movement?”. Participants were instructed before the experiment began to focus on how much they liked watching each movement, on a 9-point Likert scale (1 = did not like at all; 9 = liked very much) using the corresponding keys on the computer keyboard. There was no reaction time cut-off for the ratings. The next trial began immediately after the computer registered a response.

After this rating phase, participants performed a recognition task to ensure that they had attended to the videos. Participants watched 20 dance sequences, 10 previously viewed during the main experiment and 10 novel ones. After viewing each sequence, they classified it as novel or previously viewed. This recognition task enabled us to ensure that participants had attended to the dance sequences during the task. The task was programmed using the Psychophysics toolbox [32–33] for MATLAB (v7.2; MathWorks, Natick, MA) and presented on a computer running Windows XP.

### Questionnaires

Participants completed the Interpersonal Reactivity Index (IRI) to assess empathy, or the degree to which participants are sensitive to others’ affective experiences [34–35]. The IRI consists of 28 questions, assessed on 5-point scale (A = does not describe me well to E = describes me very well). The questionnaire includes four sub-scales, each with seven items. These consist of: *fantasy* (FS), the ability to identify with fictional characters; *perspective-taking* (PT), the extent to which individuals spontaneously adopt others' viewpoints; *empathetic concern* (EC), individuals' feelings of warmth, compassion, and concern for others; and *personal distress* (PD), participants’ feelings of distress at another's negative experience.

In addition to the IRI, dance experience and other basic demographic information, participants completed a two-item mood questionnaire, assessing general emotional state after viewing the video clips (anchors: 1 = very bad; 5 = very good; for non-dancers: *M* = 3.77; *SD* = 0.71; dancers: *M* = 4.08; *SD* = 0.57) and the extent to which their current mood was related to the dance movements (anchors: 1 = not at all; 5 = completely; for non-dancers: *M* = 2.12; *SD* = 1.21; dancers: *M* = 2.52, *SD* = 1.12) on a 5-point Likert scale. This questionnaire confirmed that there were no differences between dancers and non-dancers in terms of mood, *t*(49) = -1.717, *p* = 0.092, *d* = .49, or global affect, *t*(49) = -1.236, *p* = 0.222, *d* = .35, induced by the experiment.

### Facial electromyography recording

Facial muscle activity was recorded from the *zygomaticus major* (cheek) and *corrugator supercilii* (brow) muscles on the left side of the face using the anatomical guidelines described in Fridlund and Cacioppo [36], during the passive viewing phase of the study. We used 4mm diameter, shielded, Ag/AgCl surface electrodes placed in pairs (1cm inter-electrode distance) oriented parallel to the muscle fiber bodies, linked to a single reference electrode in the centre of the forehead near the hairline. To reduce impedance, electrode cups were filled with conductive gel before placement and skin surfaces were cleansed and then scrubbed with a mildly abrasive pad coated with a thin film of electrode gel [36]. After electrode placement, participants completed the Interpersonal Reactivity Index (IRI) questionnaire [34–35] as a measure of interpersonal empathic capacity and to allow the electrodes time to reach recording equilibrium.

A BIOPAC MP36 system connected to a computer running BIOPAC’s AcqKnowledge Software detected and amplified the EMG signals. This system uses a “common mode rejection” algorithm, which subtracts signal at the reference site from signal at the recording sites to remove environmental noise. We sampled at a rate of 2000Hz, with a gain of 5000 and applied a 50Hz notch filter to minimize electrical noise. With the exception of the notch filter, all signal frequencies ranging from 0.5Hz to 500Hz were amplified and recorded for offline analysis.

### Signal Processing

To process the EMG data, the data were first bandpass filtered using a fourth-order Butterworth filter (10-400Hz). We used 400Hz as our low-pass cut-off because frequencies greater than 400Hz contribute negligibly to facial EMG signals [37]. Trials in which participants blinked during the presentation of the video or produced large facial movements unrelated to the task (e.g., yawns, coughing) were excluded. Data were then full-wave rectified and smoothed (using a gaussian smoothing kernel-transformation over the data, with FWHM = 70.645). The pre-stimulus baseline was computed over the 500ms preceding the video onset. We normalized each event by the computed baseline, dividing the EMG signals during the video by the computed baseline, to allow comparisons across participants and conditions [38]. All EMG data were processed and analysed using purpose-written functions in MATLAB (The Mathworks, Natick, MA).

### EMG Data analysis

To explore the relationship between facial EMG signals and participants’ subjective liking ratings, we examined participants’ ratings for each video. We split the 40 video ratings into 3 categories for each participant, based on each participants’ own liking ratings. In order to have equivalent numbers of videos for each participant in the main experimental categories of interest (like/dislike), videos were ordered based on liking ratings and were then split so that the 15 videos with the lowest ratings were assigned to the ‘dislike’ category, the 15 videos with the highest ratings were assigned to the ‘like’ category, and the 10 remaining videos were assigned to a ‘neutral’ category, for each participant. This allowed us to ensure that the videos we used for each participant reflected particular participants’ affective experience, rather than more general ratings. This is a critical step in linking subjective experience, which differs across participants, with facial behavior.

Our analyses focused on comparisons between the like and dislike categories, as our *a priori* expectation was that neutral videos should evoke little affective experience. The average rating for disliked videos was 2.62 (*SD* = 0.77; range from 1.28 to 4.17) and liked videos was 6.99 (*SD* = 0.86; range from 4.5 to 8.83). It is important to note that no differences emerged between dancers’ and non-dancers’ average ratings for both categories (dislike: non-dancers: *M* = 2.47 *SD* = 0.76, dancers: *M* = 2.76 *SD* = 0.79; like: *M* = 6.82 *SD* = 0.67, dancers: *M* = 7.15 *SD* = 1.02; no differences between the two groups, all *p* values > 0.2). The EMG signal was then averaged across the whole video, as we did not have *a priori* expectations about the time course of activity. We averaged the EMG signal for each video across all three passive viewing trials of that video (Part 1). We then computed the grand average of liked and disliked videos for each participant.

The main analysis consisted of a mixed-model ANOVA with average EMG amplitude as the dependent variable, video category (liked versus disliked) as the within-subjects factor and dance experience (dancer versus non-dancer) as the between-subjects factor, for each muscle. This enabled us to explore potential differences in EMG signal between liked and disliked videos, and whether dance experience impacts this difference.

## Results

**Fig 2:**
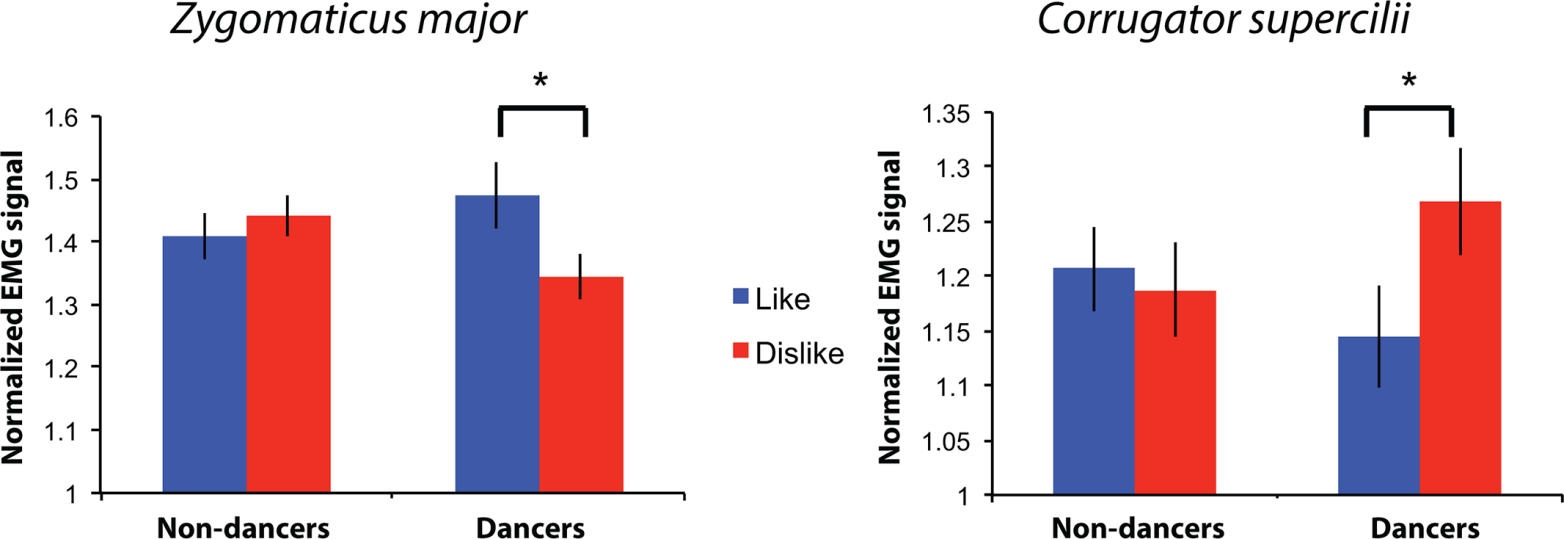
Normalized EMG signal, averaged across the 3s videos, by muscle, reported valence and group. The left panel illustrates the EMG signal from the *zygomaticus major* (ZM) muscle and right panel for the *corrugator supercilii* (CS) muscle. Blue bars indicate muscle activity averages for liked videos, red bars for disliked videos. An asterisk (*) indicates a difference significant at *p* < 0.05. Error bars indicate +/- 1 standard error of the mean (SEM).

**Fig 3:**
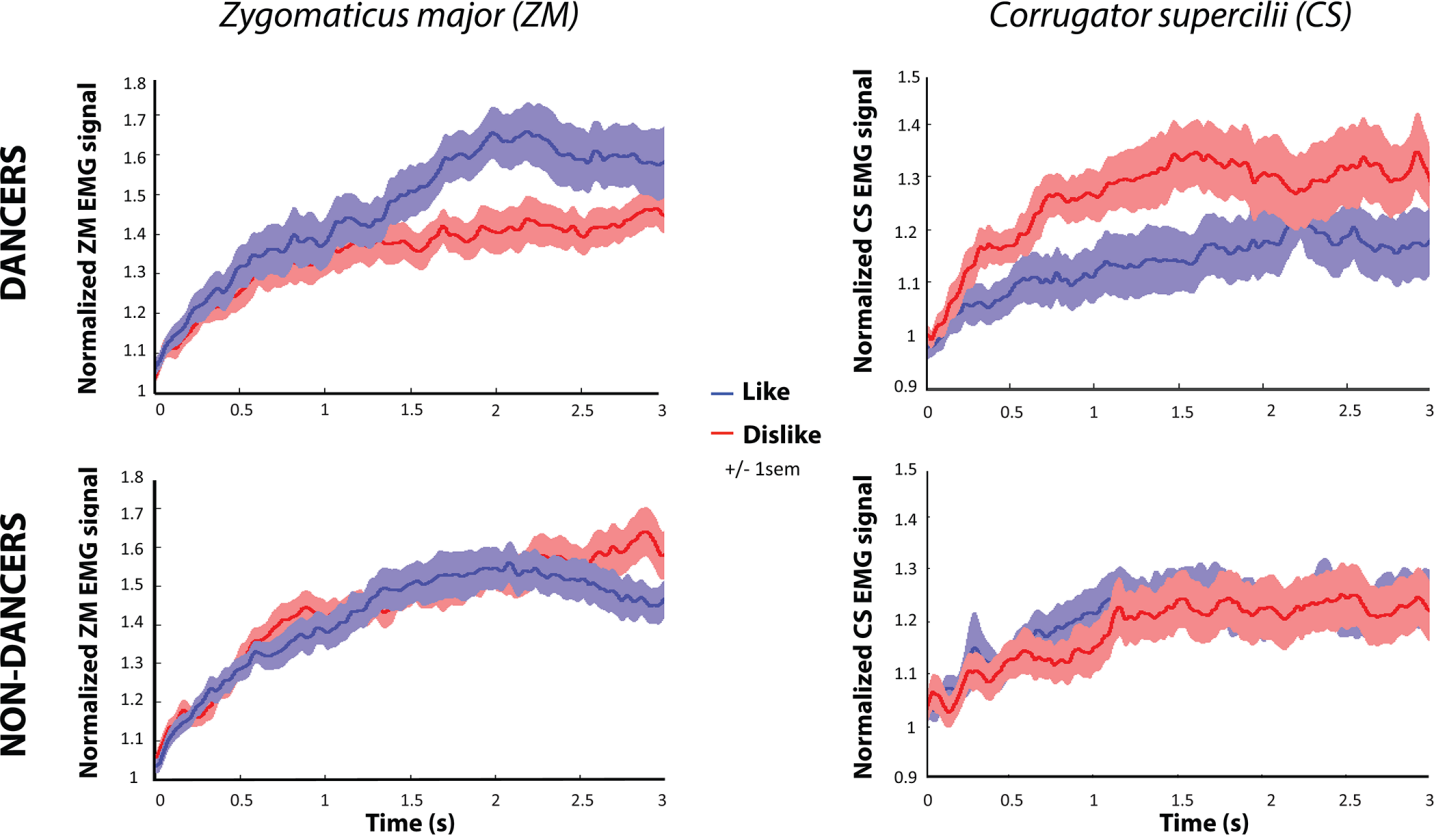
EMG activity across time. Graphs depict changes in EMG traces in standardized units for dancers and non-dancers, for each muscle (*zygomaticus major* and *corrugator supercilii*), depending on participants’ subjective liking rating (like/dislike). Shaded regions show +/- 1SEM. For illustration purposes, the condition hypothesized to show a greater response has been visualized on top of the other condition (i.e., blue traces for ZM activity and red traces for CS activity).

We predicted more activity in the ZM muscle when observing liked videos compared to disliked videos and the inverse relationship for the CS muscle. We also hypothesized that prior experience would increase implicit liking of observed movements. We therefore expected to find an interaction between dance experience and EMG amplitudes for liked and disliked movements. Our data support this hypothesis by demonstrating a significant interaction between liking category (like/dislike) and dance experience for both the ZM muscle, *F*(1,49) = 4.796, *p* = 0.033, η^2^_p_ = 0.089; and the CS muscle, *F*(1,49) = 4.438, *p* = 0.040, η^2^_p_ = 0.083 (Fig 2). There was no main effect of liking a video on the EMG signal: ZM: *F*(1,49) = 1.796, *p* = 0.186, η^2^_p_ = 0.035; CS: *F*(1,49) = 2.387, *p* = 0.129, η^2^_p_ = 0.046; nor was there a main effect of dance experience on the EMG signal: ZM: *F*(1,49) = 0.108, *p* = 0.744, η^2^_p_ = 0.002; CS: *F*(1,49) = 0.031, *p* = 0.861, η^2^_p_ = 0.001 (see S1 Text for further time course analyses of CS and ZM activity–Fig 3).

To determine the source of the interaction, we examined EMG signal differences between liked and disliked videos for dancers and non-dancers separately. Results showed a significant difference for dancers only, for both muscle sites (Dancers: ZM: *t*(24) = 2.124, *p* = 0.044, *d* = 0.42; CS: *t*(24) = -2.543, *p* = 0.018, *d* = 0.51; Non-dancers: ZM: *t*(25) = -0.747, *p* = 0.462, *d* = 0.09; CS: *t*(25) = 0.403, *p* = 0.690, *d* = 0.17; see S2 Text for further analysis of sex and age effects on ratings and EMG activity). As these analyses show, and as the average values in Fig 2 and the time series in Fig 3 show, facial muscle activity during dance sequence observation was indeed influenced by liking ratings and prior dance experience: we observed a higher CS activity for disliked videos and inversely an higher ZM activity for liked videos, only among experienced dancers. Importantly, differences in participants liking ratings did not account for EMG activity differences, as there were no differences in the average liking ratings across the participants (*M*_*non-dancers*_ = 4.63, *SD*_*non-dancers*_ = 0.66; *M*_*dancers*_ = 4.97, *SD*_*dancers*_ = 0.86; *t*(49) = -1.553 *p* = .127, *d* = 0.44). Similarly, no difference of variance in ratings between dancers and non-dancers was present (F(1,50) = 0.412, *p* = 0.524). Moreover, a strong correlation between average rating per video made by dancers and non-dancers was found (r = 0.919, *p* < 0.001), suggesting broad consensus between dancers and non-dancers in terms of how much each video was liked.

One possible explanation for the interaction that emerged between dance experience and EMG signal may be that differences in perceptual fluency drive differences in attention to the stimuli across the groups. However, we found no differences between dancers and non-dancers in recognition accuracy in the post-experiment attentional task, *t*(49) = -0.744, *p* = 0.460, *d* = 0.20, suggesting that both groups found the videos similarly memorable (*M*_*non-dancers*_ = 0.88, *SD*_*non-dancers*_ = 0.08; *M*_*dancers*_ = 0.90, *SD*_*dancers*_ = 0.1).

A second alternate explanation for our group differences in EMG activity may be that performing artists differ from non-artists in their empathic responses to others. That is, dancers may be more sensitive to the stimuli. To assess this idea, we used independent samples t-tests to compare dancers to non-dancers on the IRI subscales. We found no differences between the groups on any IRI subscale, meaning that differences in affective reactivity between the groups on the task are unlikely to be due to differences in empathic capacity, as measured by the IRI (fantasy: *t*(49) = 0.516, *p*_*uncorrected*_ = 0.608, *d* = 0.14; perspective taking: *t*(49) = 0.308, *p*_*uncorrected*_ = 0.759, *d* = 0.09; empathetic concern: *t*(49) = -1.680, *p*_*uncorrected*_ = 0.099, *d* = 0.47; personal distress: *t*(49) = -1.570, *p*_*uncorrected*_ = 0.123, *d* = 0.44).

Finally, to rule out the possibility that differences between the two populations were due to differences in their perceptions of the emotionality expressed in each movement, we conducted a follow-up online study with two independent samples of participants. These included a non-dancer group that comprised individuals with no dance experience (*N* = 20; 14 females, *M*_*age*_ = 19.6 years, *SD* = 0.99); and a dancer group, comprising individuals who had general dance experience (*N* = 15; 15 females, *M*_*age*_ = 22.3 years, *SD* = 4.73). Participants rated each stimulus, using a 9-point Likert scale (anchors: 1 = not at all; 9 = a lot/very much), on the extent to which the dancer expressed emotion through his or her movement. An independent samples t-test on the average ratings made by dancers and non-dancers confirmed that the findings were not attributable to differences in emotional ratings across the groups, *t*(33) = 0.076, *p* = 0.940, *d* = 0.026. These data demonstrate that dancers and non-dancers perceived the videos to be similarly emotionally evocative. We are therefore confident that the differences between dancers and non-dancers are not solely attributable to differences in how they view the stimuli (for further details on the follow-up study, see S3 Text and S1 Table).

### The role of specific dance experience in EMG responses

**Fig 4:**
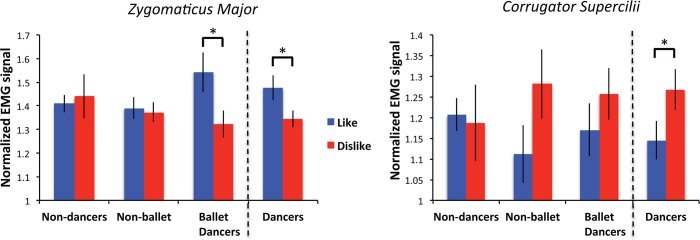
ZM and CS EMG signal by participant group: Non-dancers vs. non-ballet dancers vs. ballet dancers. These findings illustrate that the EMG signal of ZM was greater for liked videos among ballet dancers only. Error bars show +/- 1SEM and an asterisk (*) denotes a difference significant at *p* < 0.05.

Our final analysis aimed to further investigate the relationship between action experience and affective responses by probing the extent to which the richness of an observer’s prior dance training experience shapes affective responses. If, as we have argued, specific training experience does indeed shape EMG responses to the videos, then dancers with more ballet experience should show more pronounced effects, relative to dancers without ballet experience, as all the stimuli in the experiment featured ballet movements (ranging from classical to modern ballet). To test this hypothesis, we split the dancers into 2 groups: non-ballet dancers with less than 6 months of ballet experience (*N* = 11) and experienced ballet dancers with more than 2 years of ballet experience (*N* = 14; for more details see Table 1), and subjected the EMG data from just these individuals to similar ANOVAs as above. As expected, ballet experience significantly influenced ZM activity, *F*(2,48) = 4.365, *p* = 0.018, η^2^_p_ = 0.154, such that ballet dancers showed more ZM activity for liked movements than non-ballet dancers. However, this effect only emerged at the trend-level for CS activity, *F*(2,48) = 2.542, *p* = 0.089, η^2^_p_ = 0.096 (Fig 4). This finding corroborates the main study findings and provides additional support for the notion that experience with a particular movement vocabulary can shape physiological responses to stimuli under passive observation conditions above and beyond the influence of dance training more generally.

## Discussion

Through the present study, we aimed to address two complementary questions. First, we sought to determine whether affective evaluation of non-emotional stimuli shapes facial behavior as measured by EMG. Second, we asked whether prior experience with complex whole-body movements influences this effect. Results from facial EMG demonstrate that experienced observers show significantly greater CS activity when watching disliked movements compared to liked movements. We found the inverse pattern for the ZM muscle (increased ZM activity for liked versus disliked movements). Among non-dancers, however, this pattern did not reliably emerge in either muscle. These findings suggest that facial muscles subtly echo the affective evaluations of experienced observers during complex action observation, but such a pattern is absent among inexperienced observers. These findings underscore the potential of EMG as method to implicitly examine subtle shifts in affective responses evoked by artistic performances, as well as stimuli that are not necessarily (or intentionally) emotional in nature.

### From Affect to Aesthetics

In recent years, research into the neuroaesthetics of movement has gained momentum as more investigators probe the relationship between brain processes and aesthetic experiences among those watching dance [1, 19, 21–25, 39–40]. Affective judgment, liking and aesthetic experience are closely related and we can consider that asking a participant how much he or she likes watching a movement is similar to asking about his or her aesthetic experience with the movement [19, 39]. In relation to prior research on neuroaesthetics [19–21, 39], our findings provide an alternative perspective on the role of implicit reactivity when watching dance. While most previous studies have included an explicit rating task in their experimental design [19–21], in the present study we measured implicit affective evaluations prior to informing participants that they would be asked to make explicit ratings. Thus, we measured implicit affective responses during simple observation and show that dance-experienced observers produce arguably more spontaneous affective responses that correspond to their explicit aesthetic evaluation during action observation. As such, this finding stands in contrast with suggestions by previous literature that aesthetic processing requires intention and is not spontaneous in character [41–42]. Previous research has demonstrated that when expert observers watch another person perform familiar actions, experts’ sensorimotor cortices are more engaged compared to naïve observers [43–44]. Moreover, such sensorimotor cortical engagement is also closely associated with an observer making a positive affective/aesthetic judgment about an observed movement [19, 21, 39]. In the present study, it is likely that non-dancers did not affectively engage with movements with which they had little prior visuomotor experience. This may explain why activity from the ZM muscle for liked movements was obtained for dancers only, and those with more ballet experience specifically.

Our results thus support an embodied account of affective judgment, in accord with Freedberg and Gallese’s theory [45]. This theory states that an important factor in shaping an observer’s aesthetic experience is the simulation of actions and emotions visible or implied in an artwork. Considering this theory in light of our results, it seems that sensorimotor experience plays a crucial role in the creation of measurable implicit affective reactions to observed movements, as those without experience may lack the expertise to engage in motor simulation, despite being able to appreciate the aesthetic value of a movement at the explicit level. The follow-up questionnaire performed with an independent sample of experienced dancer and non-dancer participants sheds further light on this relationship, suggesting that experienced dance observers report overall higher ratings of liking and perceived reproducibility for the stimuli examined in the present study (see S3 Text and S1 Table).

### Affective Responses to Whole-Body Actions

It is important to note that, to our knowledge, this is the first time facial EMG has been used to study affective responses to non-explicitly valenced, dynamic whole-body movement. Most prior work has focused on static images and faces [8, 11, 31]. Indeed, only one study to date has examined whole body actions–however, these actions were explicitly expressing anger [18]. The fact that we were able to measure affective responses to non-intentionally emotionally valenced whole-body action stimuli suggests that watching actions can elicit physiological responses that provide insights into an observer’s affective experience of a particular movement. It is possible that if we had used intentionally emotionally-valenced dance stimuli instead, this might have amplified effects among all participants. However, stimuli that are clearly emotionally-valenced might have also obscured any differences based on sensorimotor experience between dancers and non-dancers. Instead, the movements featured in the present study were not intentionally emotionally valenced and were not performed to elicit a particular emotion in the observer, thus allowing us to more sensitively examine the impact of implicit emotional engagement.

Previous literature on facial mimicry suggests that participants mimic the mannerisms, facial expressions, and emotions of those they observe [14, 46–48]. However, the dancers featured in our stimuli were filmed at a distance where participants could not identify facial expressions or emotion on the dancers’ faces. Moreover, we explicitly asked the dancers to maintain a neutral facial expression when we were filming the stimulus videos, meaning that dancers’ facial expressions should not have been conveying any obvious affective cues (and indeed, the post-study follow up questionnaire we administered to a new set of participants confirmed that this was generally the case, see S3 Text and S1 Table). Another argument against the possibility of facial mimicry explaining our results is that if mimicry were driving our findings, we have no *a priori* reason to expect dancers to engage in more facial mimicry than non-dancers–indeed, facial mimicry is commonly observed in response to emotional faces [13–16]. Our findings are thus more consistent with an embodiment account of facial reactivity during action observation than a mimicry account. We acknowledge that differences in attention or interest in the stimuli between dancers and non-dancers remains a potentially confounding factor, although data from our memory task suggest that both groups attended to the stimuli in similar fashion.

### Prior Experience and Perceptual Fluency

Another theoretical account that both informs and is informed by the present findings is Winkielman and Cacioppo’s model of hedonic fluency [11]. This model asserts that the experience of fluency produces positive affective experiences in individuals. In support of this model, previous work demonstrates that people like stimuli they find easy to understand or interact with more than awkward, overly complex or difficult to understand stimuli [49–51]. Considering the present results in light of this model, it seems plausible that specific experience with the dance movements leads to greater or less fluent processing (depending on the specific movements), and this is reflected by more pronounced positive or negative affective responses in the experienced observers only (see also [11, 31, 52–53]). It is of note that the present findings are the first to suggest a direct relationship between prior experience with an action and its affective value, as evidenced by facial muscle responses, and myriad possibilities exist for deeper investigation into the relationship between embodiment, fluency, and affect.

Because participants were blind to the study aims and had no knowledge of the explicit rating task they would perform during the final portion of the study, we can be confident that the implicit emotional responses we detected in the first block of trials were not affected by task demands. An important point highlighted by our results is that implicit/objective and explicit/subjective affective reactions are not necessarily correlated and ‘automatic’, as we found correspondence between implicit (EMG signal) and explicit (liking ratings) only among dancers. This finding suggests that an observer needs some degree of prior experience with a movement in order to understand that movement and produce an affective facial reaction. However our design does not allow us to test whether it is specifically visual or physical experience that influences the pattern of facial responses reported here. Futures studies could address this question by including objective physical performance measures, or by adding an additional group of participants who are experienced dance spectators who have not received any formal dance training. Alternatively, future work could also adopt the approach used by Kirsch and colleagues [20–21] that involves training novice participants to perform complex dance sequences under different training conditions (i.e., visuomotor vs. visual only), and assess the impact of these types of experience on implicit affective responses during action observation.

One final point raised by the current study that is worth considering is what we are actually measuring or assessing when we ask both experienced and novice participants to rate how much they “like” a given dance stimulus. One possibility is that observers judge technical proficiency or virtuosity of the observed dancer. This could in principle explain why the novice sample in the present study did not show a difference in facial muscle activity, because these dance-inexperienced individuals may not be able to tell which dance moves were performed particularly well and which were less technically accurate. To address this possibility, follow up work could directly assess the relationship between an observer’s prior visuomotor experience, ratings of technical proficiency of individual movements, and liking ratings.

## Conclusion

We report evidence for a relationship between aesthetic evaluations, affective responses, and an observer’s prior sensorimotor experience, as evidenced by observers’ facial muscle responses when watching dance movements. Results from dance-experienced observers showed relatively greater responses in CS muscle activity for disliked movements and in ZM muscle activity for liked movements. Closer examination of the data demonstrates that the ZM finding was driven by ballet-experienced participants, who showed this dissociation between affective valence and EMG activity for the ZM muscle to a higher degree than dancers who do not have any prior ballet training. The relationship between the explicit ratings and EMG data in experienced observers suggests that facial muscles sensitively echo affective judgments, thus underscoring the potential of EMG as a method to examine subtle shifts in implicit affective responses evoked by performance art. The present study should also help clarify how sensorimotor and affective systems interact to shape people’s responses when watching dance. These findings will also be of interest to professional choreographers, who may wish to design works that evoke certain emotions among observers by planning specific choreographic choices depending on the expected experience of audience members. Finally, the methodological approach and findings from this study have implications for assessing somatic responses to dynamic stimuli across a broad range of applied contexts, from advertising and marketing to the psychology of art perception.

## Supporting Information

S1 Table Follow-up questionnaire findings on relationship between experience, embodiment and affective evaluation. (DOCX)

S1 Text Time course analysis for CS and ZM muscle activity. (DOCX)

S2 Text Analyses of sex and age effects on ratings and EMG activity. (DOCX)

S3 Text Follow-up questionnaire findings on relationship between experience, embodiment and affective evaluation. (DOCX)
