# Supplementary material for: The Impact of Experience on Affective Responses during Action Observation
Source: PLoS One. 2016 May 5;11(5):e0154681. doi: 10.1371/journal.pone.0154681 (PMC4858140; doi:10.1371/journal.pone.0154681)
Supplement: S1 Table — (DOCX) [file pone.0154681.s001.docx]

**S1 Table**

**Data from the online questionnaire for stimulus validation - the data/results presented in this table are detailed in S3 Text**

**Supporting Information Table 1.** Correlations between participants’ arousal, actor’s arousal, movement’s smoothness, participant movement’s liking and movement reproducibility.

|  | **Actor's arousal** | **Movement smoothness** | **Participant movement’s liking** | **Movement’s reproducibility** |
| --- | --- | --- | --- | --- |
| **Participant's arousal** | rND = .850***;  rD = .872*** | rND = .365*;  rD = .571*** | rND = .731***;  rD = .851*** | rND = -.785***;  rD = -.738 *** |
| **Actor's arousal** |  | rND = .483**;  rD = .603*** | rND = .774***;  rD = .883*** | rND = -.843***;  rD = -.718*** |
| **Movement smoothness** |  |  | rND = .544***;  rD = .688*** | rND = -.280;  rD = -.256 |
| **Participant movement’s liking** |  |  |  | rND = -.786***;  rD = -.791*** |

*: p<0.05; **: p<0.01; ***: p<0.001; rND = pearson correlation factor for non-dancers; rD = pearson correlation factor for dancers.
