# Supplementary material for: The Impact of Experience on Affective Responses during Action Observation
Source: PLoS One. 2016 May 5;11(5):e0154681. doi: 10.1371/journal.pone.0154681 (PMC4858140; doi:10.1371/journal.pone.0154681)
Supplement: S1 Text — (DOCX) [file pone.0154681.s002.docx]

**S1 Supporting Information**

**Time course analysis for CS and ZM muscle activity**

The interested reader might be interested in the time course of the effect illustrated in Fig 3, as this figure suggests a difference between the time course of activity for ZM and CS muscles. We therefore split the EMG signal over the 3-second video into six 500ms time windows, and conducted a similar ANOVA analysis, adding time as an extra within-subjects variable. For the CS muscle, none of the interaction effects reached significance (p-values > 0.164). However the ZM muscle tended to show a liking x time interaction (*p* = 0.069) such that the dissociation between liked and disliked EMG activity was present only in a later phase. There was also a dance experience x liking x time interaction at the trend level (*p* = 0.064). This showed that dance experience had a trend effect on the interaction between liking and time course of the ZM, as observed in Fig 3.
