# Supplementary material for: The Impact of Experience on Affective Responses during Action Observation
Source: PLoS One. 2016 May 5;11(5):e0154681. doi: 10.1371/journal.pone.0154681 (PMC4858140; doi:10.1371/journal.pone.0154681)
Supplement: S2 Text — (DOCX) [file pone.0154681.s003.docx]

**S2 Supporting Information**

**Analyses of sex and age effects on ratings and EMG activity**

1. **Sex difference analyses**

The dancer group included no male participants whereas the non-dancer group included 10 male participants. We therefore examined the differences between male and female non-dancers on several aspects of the data using ANOVAs similar to those reported in the main text. Our results show that there were no differences between male and female participants in terms of how they rated the videos (*p* = 0.53), or in EMG activity for the ZM or CS muscles for liked or disliked movements (all *p* values > 0.30). These data suggest that sex differences are unlikely to be the primary driver of the results.

Moreover, we have examined the data from only female non-dancers (as our experienced dancer sample comprises females only). When we rerun our analyses with only female non-dancers (which naturally reduces the power of these analyses), we find no difference between the EMG response during liked and disliked videos for both CS and ZM (CS: t(15) = -0.324, *p* = 0.750; ZM: t(15) = -0.260, *p* = 0.798). When rerunning the overall analysis on these unbalanced groups, the interaction between dance experience and liking no longer reaches significance (CS: F(1,36) = 1.868, *p* = 0.180; ZM: F(1,39) = 2.667, *p* = 0.111).

1. **Age difference analysis**

Because the experienced dancer group was on average slightly older than the non-dancer group, we examined correlations between age, liking ratings, and EMG activity. These results show that age did not have a significant impact on liking ratings (*p* =0.39); or EMG activity for either ZM or CS muscles for liked or disliked movements (all *p* values > 0.70). These extra analyses confirm that demographic differences between our groups are unlikely to have accounted for the results.
