# Supplementary material for: The Impact of Experience on Affective Responses during Action Observation
Source: PLoS One. 2016 May 5;11(5):e0154681. doi: 10.1371/journal.pone.0154681 (PMC4858140; doi:10.1371/journal.pone.0154681)
Supplement: S3 Text — (DOCX) [file pone.0154681.s004.docx]

**S3 Text**

**Stimulus validation via online questionnaire**

In order to probe in more detail what emotional content participants saw in the stimuli and how the stimuli impacted their own emotional state, we conducted an online questionnaire using Qualtrics^TM^ with two groups of participants who did not participate in the main study: a non-dancer group (ND) that comprised individuals with no dance experience (N=20); and dancer group (D), comprising individuals who were experienced in dance in general (N=15^[[1]](#footnote-1)^).

We aimed to test four specific questions, namely: (1) how the movement featured in the stimuli changes the emotional state of the observer (subjectively); (2) how the observer perceives the emotional state of the dancer as expressed via the dancer’s movement; (3) how smooth or awkward the movements were perceived as; and finally (4) how well the observer perceived his or her ability to reproduce the movement performed by the dancer.

Participants were asked to rate each stimulus on the following questions, using a 9-point Likert scale:

(1) - How joyful does this movement make you feel? (JOYFUL)

- How excited does this movement make you feel? (EXCITED)

- How bored does this movement make you feel? (BORED)

(2) - How happy do you think the dancer is? (HAPPY DANCER)

- How excited do you think the dancer is? (EXCITED DANCER)

- How bored do you think the dancer is? (BORED DANCER)

- How sad do you think the dancer is? (SAD DANCER)

(3) - How awkward do you think this movement looks? (AWKWARD)

- How smooth do you think this movement looks? (SMOOTH)

(4) - How well could you reproduce this movement?  (REPRODUCE)

**Data analysis and Results**

- *Grouping questions in 3 categories: (1) Observer’s arousal (joyful, excited and bored); (2) Actor’s arousal (happy, excited, bored, sad dancer) and (3) movement trait (smooth and awkward).*

We inverted participants’ responses to negative questions (bored, sad and awkward), to create a consistent set of responses in each category. We calculated Cronbach's alpha for each category, for non-dancers and dancers respectively (observer’s arousal: αND=.926; αD=.938; actor’s arousal: αND=.901; αD=.932; movement trait: αND=.811; αD=.910). As all α - values are greater than 0.8, we can be confidant that our categories are homogeneous and that we can group the items together into each category.

- *Correlations between category ratings (averaged per video per participant group) and averaged liking ratings from the EMG experiment (taken from non-dancers and dancers respectively).*

As mentioned in the main text, we have used the same stimuli for several experiments and pilot studies in the past (Cross et al., 2011; Kirsch & Cross, 2015). We consistently find high inter-subject correlations of liking ratings made by different groups of participants. As such, it would appear that average population ratings for liking are stable for this set of stimuli.

***Interpretation:*** Overall, as presented in S1 Table, we found a strong relationship between affective judgment (as assessed by liking ratings), and emotional state of the observer and of the observed actor. Results show as well that liking judgments were correlated with a movement’s characteristics, with participants preferring smooth movements. Moreover, a movement’s reproducibility correlated reliably with the observer’s and actor’s arousal. Movements that were rated as more reproducible were associated with lower arousal in participants and actors. However, movement’s reproducibility did not correlate with a movement’s smoothness. Overall these results suggest that the stimuli elicited some emotional arousal in observers, which related to the perceived arousal of the dancer, as well as how much the participant liked the movement. This pattern of findings was present for both dancers and non-dancers, making it less likely that it is these relationships that underpin the EMG effects reported in the main experiment.

- *Evaluating differences between dancers’ and non-dancers’ ratings for each question category*

To determine whether dancers and non-dancers differ in their rating responses to each main category, we performed independent samples t-tests on the average rating per video made by dancers and non-dancers. We found a significant difference between dancers’ and non-dancers’ ratings for each category, except for actor’s arousal:

- observer’s arousal: t(39) = -3.970, p < 0.001
- actor’s arousal: t(39) = .430, p = .670
- movement trait: t(39) = -4.813, p < 0.001
- movement’s liking: t(39) = -2.670, p = 0.011
- reproducibility: t(39) = -18.067, p < 0.001

***Interpretation*:** It appears that dancers report higher personal arousal than non-dancers overall, and liked watching the movements more. Moreover, they perceived the movements as smoother and more natural than non-dancers. Not surprisingly, dancers also perceived their ability to reproduce the observed movement as significantly better than non-dancers (and recent work corroborates the relationship between perceived and actual ability to reproduce observed complex actions; Kirsch et al., 2015). However, the data demonstrate that both dancers and non-dancers perceived the dancers to be displaying similar levels of arousal. As such, it does not seem that in the present study any differences exist between dancers and non-dancers in terms of perceiving an emotional state in an observed other. Instead, dancers’ personal experience of watching dance movements is quantitatively different than that of non-dancers. This finding suggests a sensorimotor effect of prior experience might underpin the EMG results from the main experiment. In other words, the dancers we tested in both the on-line survey and the main EMG study seem to be more receptive and expressive of emotions that non-dancers when they watch dance movements.

1. For full transparency, and due to a limited pool of experienced dancers, two dancers who participated in the main experiment also participated in the online questionnaire. [↑](#footnote-ref-1)
